# Physiological Regulation in Young Children During Parent–Child Free Play: Attachment-Related Differences and RMSSD Synchrony

**DOI:** 10.3390/bs16050739

**Published:** 2026-05-09

**Authors:** Hyo Jeong Jeon, Eun-Kyoung Goh

**Affiliations:** 1 Department of Child Studies, Dong-A University, Busan 49315, Republic of Korea; hjeon@dau.ac.kr; 2 Human Life Research Center, Dong-A University, Busan 49315, Republic of Korea

**Keywords:** attachment security, physiological regulation, heart rate variability, parent–child interaction, physiological synchrony

## Abstract

This study examined parent–child physiological synchrony within the context of interactions and attachment-related differences. Specifically, this study investigated physiological synchrony, as indexed by the association between parent and child root mean square of successive differences (RMSSD) during free-play interactions, and differences in children’s mean heart rates according to attachment classification. The participants were 25 parent–child dyads (mean child age = 36.48 months). Physiological responses were assessed during free-play interactions using heart rate (HR) and heart rate variability (HRV). Children’s attachment was classified as secure or resistant based on their behaviors observed during the separation–reunion procedure. The results showed a significant positive association between the parent and child RMSSD (ρ = 0.48, *p* < 0.05). Parental anxiety was positively associated with both parents’ and children’s physiological arousal. Attachment-related group differences were observed only in the mean heart rate, with children with resistant attachment showing a significantly higher HR than those with secure attachment (t = 2.69, *p* < 0.05). No significant group differences were observed in the RMSSD or HR/RMSSD ratios. Overall, these findings suggest that the parent–child RMSSD association, as a component of physiological synchrony, may reflect a normative feature of parent–child interaction that emerges across attachment classifications. In addition, attachment-related differences were primarily observed in physiological arousal.

## 1. Introduction

Early social–emotional development is fundamentally embedded within parent–child relationships, where children’s capacities emerge through repeated interactions with caregivers. Increasing attention has been directed toward dyadic processes that capture how parents and children coordinate their behavioral, emotional, and physiological states during their interactions. Among these processes, synchrony, defined as the temporal coordination of activity between interaction partners, has been identified as an important mechanism underlying early development (Feldman, 2007, 2012; Harrist & Waugh, 2002). Rather than conceptualizing regulation solely as an individual capacity, this perspective emphasizes that children’s functioning is shaped by ongoing interactions, highlighting the central role of dyadic processes in development.

A key extension of this perspective is that synchrony operates not only at the behavioral level but also at the physiological level. Feldman (2007, 2012, 2017) demonstrated that biobehavioral synchrony emerges from early parent–infant interaction and plays an important role in the development of self-regulation and social functioning. Empirical findings further show that parents and infants coordinate physiological signals such as heart rhythms during interactions (Feldman et al., 2011). Notably, this synchrony was observed prior to the establishment of stable attachment classifications, suggesting that it reflects an early emerging relational process rather than being merely an outcome of attachment. Taken together, these findings indicate that physiological synchrony contributes to the early formation of attachment relationships in infants.

Importantly, synchrony is not limited to infancy but continues across later developmental stages. Studies on toddlers and preschool-aged children have demonstrated that physiological synchrony can be observed in naturalistic contexts such as free play and structured interaction (Davis et al., 2018; Lunkenheimer et al., 2015). These findings suggest that synchrony is a common feature of parent–child interactions across development. Moreover, synchrony has been linked to children’s socioemotional functioning, supporting its relevance as a developmental process rather than a stage-specific phenomenon.

Beyond parent–child relationships, synchrony has been associated with broader social bonding processes. For example, the physiological synchrony observed during initial interactions between adults has been linked to interpersonal attraction and partner selection (Zeevi et al., 2022). Although these findings are not specific to parent–child relationships, they support the idea that synchrony may reflect a general mechanism involved in the formation of social connections.

Within this framework, the autonomic nervous system provides a key biological basis for understanding emotional and relational processes. Heart rate variability (HRV), particularly the root mean square of successive differences (RMSSD), reflects parasympathetic activity and is widely used as an index of physiological flexibility (Cattaneo et al., 2021; Porges, 2007; Thayer & Lane, 2009). Lower HRV has been associated with greater difficulty in emotional responses, whereas higher HRV reflects better adaptive functioning (Cattaneo et al., 2021; Williams et al., 2015). In addition to HRV, the mean heart rate is an important index of physiological arousal, reflecting overall autonomic activation. Previous studies have shown that children exhibit increased physiological arousal during separation from caregivers, as reflected in changes in autonomic nervous system activity (Hill-Soderlund et al., 2008; J. D. Smith et al., 2016). However, the findings regarding differences in heart rates across attachment groups have been mixed. While some studies have reported no significant group differences, others have suggested that insecurely attached children exhibit heightened or prolonged arousal, particularly in stress-related contexts. Together, these findings indicate that heart rate (HR) may reflect attachment-related physiological functions, particularly under stress.

Attachment-related differences have been shown to be reflected in physiological functioning. Studies have reported that children’s autonomic responses vary with attachment classification, particularly in attachment-relevant contexts, such as separation and reunion procedures. For example, Hill-Soderlund et al. (2008) found differences in autonomic activation across attachment groups, and J. D. Smith et al. (2016) reported variations in parasympathetic activity and mother–child physiological associations depending on attachment patterns. Additionally, meta-analytic evidence indicates that early attachment is associated with later socio-emotional development (Groh et al., 2017). These findings suggest that attachment is linked to physiological processes, whereas synchrony reflects shared and dyadic interaction patterns.

Despite these advances, relatively few studies have examined parent–child physiological synchrony in naturalistic interactions alongside children’s physiological functioning in attachment-relevant contexts. This research gap limits our understanding of how dyadic processes observed in everyday interactions relate to children’s physiological functioning in attachment situations. To address this limitation, the present study examined (a) the association between parent and child RMSSD during free-play interactions and (b) the differences in children’s mean HR according to attachment type. By considering both synchrony- and attachment-related physiological differences, this study aims to provide a more integrated understanding of parent–child physiological processes.

Specifically, this study addresses the following research questions:

1. Is there a significant association between parent and child RMSSD during free-play interactions?
2. Do children’s mean HR differ across attachment types?

## 2. Methods

### 2.1. Participants

The sample comprised 25 parent–child dyads. The mean age of the children was 36.48 months (SD = 5.21, range = 28–47 months). The sample included nine boys (36.0%) and 16 girls (64.0%). The mean age of the parents was 37.30 years (SD = 4.14, range = 32–44). Most of the participating caregivers were mothers (n = 22, 88.0%), with a smaller proportion being fathers (n = 3, 12.0%), Table 1.

Regarding educational background, 23 parents (92.0%) had completed a college degree and two parents (8.0%) had completed high school. Household economic status was assessed via parental self-reporting and categorized as upper-middle (n = 8; 32.0%), middle-middle (n = 13; 52.0%), or lower-middle (n = 4; 16.0%).

All participants provided written informed consent after receiving a full explanation of the study procedures. The study was conducted in accordance with institutional ethical guidelines and approved by the Institutional Review Board.

### 2.2. Procedure

This study examined parent–child physiological responses and attachment-related behaviors within a controlled observational setting. Data were collected in a playroom at a community childcare support center, a public institution that provides childcare and parenting support services for young children and their families. The playroom was temporarily converted into a controlled experimental environment during the scheduled study sessions. Participants were recruited through this center, where parents of young children voluntarily enrolled and scheduled individual appointments.

All procedures were conducted by qualified personnel to ensure methodological consistency. Data collection was conducted by a licensed clinical psychologist certified by the Korean government, and behavioral observation and attachment classification were conducted by a researcher who had completed formal training in the Parent–Child Play Assessment (PCPA; Kwon & Ha, 2023).

The experimental procedure followed a structured sequence that captured the natural and context-specific responses. Upon arrival, the parents and children received a brief explanation of the study procedures and were fitted with HR-monitoring devices. A stabilization period of approximately 5 min was provided to allow participants to adapt to the recording environment, during which a picture book “Hug” (Alborough, 2000) was provided to facilitate a calm transition into the session. The playroom was arranged in accordance with the PCPA protocol, and only a designated set of play materials was used during interaction tasks.

Following this preparation, a 20 min free-play interaction was conducted, during which parents and children interacted naturally, while their physiological data were recorded simultaneously. Immediately following the free-play session, a separation–reunion procedure was administered in accordance with the PCPA protocol to observe parent–child interactions in structured conditions.

Finally, attachment-related behaviors were assessed using separation–reunion episodes under controlled conditions. The playroom was arranged to allow standardized separation procedures, including a partitioned space where the parent could leave while remaining outside the child’s view. During all procedures, only the parent, child, and experimenter were present in the room to minimize external interference. Two separation–reunion cycles were conducted, with each separation episode lasting 2 min, followed by a 3 min reunion phase. All procedures were regulated using timed auditory signals to ensure consistency across participants. Children’s behavioral responses during separation and reunion were recorded using a structured observation checklist, and attachment classifications were determined based on the standardized PCPA coding procedures.

### 2.3. Measures

#### 2.3.1. Physiological Measures

Heart rate data for both parents and children were collected using the Polar Verity Sense device (Polar Electro Oy, Kempele, Finland), a wearable photoplethysmography-based sensor that records interbeat intervals (PPI) to analyze heart rate and HRV. Based on the collected PPI data, the mean heart rate (HR; bpm), standard deviation of normal-to-normal intervals (SDNN; ms), RMSSD, and HR/RMSSD ratio were calculated. The mean heart rate was used as an index of physiological arousal, SDNN reflected overall HRV, RMSSD represented parasympathetic (vagal) activity, and the HR/RMSSD ratio was calculated to reflect increased arousal in conjunction with reduced parasympathetic regulation. To minimize the effects of initial physiological adaptation, the first 5 min of data were excluded, and the remaining 20 min collected during the free-play interaction were used for analysis.

#### 2.3.2. Play Interaction and Separation–Reunion Assessment

Parent–child interaction and attachment-related behaviors were assessed using procedures derived from the PCPA (Kwon & Ha, 2023), a standardized observational framework designed to evaluate interaction quality and attachment-related behaviors in both naturalistic and structured play contexts. The play environment and interaction tasks were organized according to the PCPA protocol. The play materials included animal figures, instrumental play items, pretend play sets (e.g., an ice cream shop and a cooking set), a rabbit family house play set, wooden blocks, LEGO blocks, and car maintenance play toys; no additional toys or external stimuli were provided. The assessment consisted of a free-play session, structured interaction tasks, and separation–reunion episodes. During the free-play session, parents and children interacted naturally using the provided materials, whereas structured play tasks were administered to observe parent–child interactions under guided conditions. Separation–reunion episodes were conducted to assess attachment-related behaviors under mildly stressful conditions.

Children’s behavioral responses during the separation–reunion episodes were recorded using a structured observation checklist, and attachment classifications were determined based on the standardized PCPA coding procedures. Attachment was initially coded according to the standard PCPA classification system, which includes secure, resistant, and avoidant categories. However, none of the children in the present sample were classified as avoidant. Therefore, for analytical purposes, attachment was categorized into two groups: secure and resistant attachment. Resistant attachment was defined as the presence of strong resistance behaviors during separation or sustained distress during reunion, whereas secure attachment was defined as the ability to tolerate separation and demonstrate relatively stable emotional regulation upon reunion. Importantly, the data used in this study consisted of HR measurements obtained from both parents and children during free-play interaction and attachment classification data derived from children’s behavioral responses during the separation–reunion procedure.

#### 2.3.3. Parental Anxiety

Parental anxiety was assessed using the Generalized Anxiety Disorder Scale (GAD-7; Spitzer et al., 2006), a 7-item self-report measure of generalized anxiety symptoms that captures key features of anxiety, including nervousness, uncontrollable worry, excessive worry, difficulty relaxing, restlessness, irritability, and fear of impending negative events. Each item is rated on a 4-point Likert scale from 0 (not at all) to 3 (nearly every day), with total scores ranging from 0 to 21, with higher scores indicating greater anxiety. The GAD-7 is considered a unidimensional measure, and only the total score was used in the present study.

### 2.4. Data Analysis

Data analyses were performed using SPSS Statistics, version 29 (IBM Corp., 2022; Armonk, NY, USA). Descriptive statistics were used to examine the distribution and central tendency of the main variables. Spearman’s rank-order correlation analyses were conducted to examine the associations between the physiological variables and parental anxiety. This nonparametric, rank-based approach was selected to account for potential non-normality in HRV data and the relatively small sample size. Independent samples *t*-tests were used to examine differences in children’s physiological indices (mean heart rate, RMSSD, and HR/RMSSD ratio) between the attachment groups (secure vs. resistant). Welch’s *t*-test was applied when the assumption of homogeneity of variance was violated, as it provides a more robust estimate under conditions of unequal variances and sample sizes. Scatterplots with fitted linear trend lines were used to visually inspect the associations between variables. All statistical tests were two-tailed, with the significance level set at *p* < 0.05, and effect sizes were calculated using Hedges’ g to account for potential bias in small sample sizes.

## 3. Results

### 3.1. Descriptive Statistics

Descriptive statistics for the main physiological variables and parent anxiety are presented in Table 2.

A total of 25 parent–child dyads were included in the analysis. The mean HR of parents was 73.75 bpm (SD = 9.79), whereas the mean HR of children was 98.57 bpm (SD = 15.50), reflecting the expected developmental pattern of higher resting HR in children than in adults.

With respect to HRV, the mean RMSSD was 276.03 ms (SD = 56.33) for parents and 254.01 ms (SD = 71.45) for children. Given that RMSSD is a commonly used index of parasympathetic (vagal) activity, these values suggest that both parents and children exhibit relatively stable autonomic regulation during free-play interactions.

The mean HR/RMSSD ratio for the children was 0.46 (SD = 0.28), indicating relatively higher physiological arousal and lower regulatory balance. The mean parental anxiety score was 3.16 (SD = 2.25).

### 3.2. Correlations Among Physiological Variables

Spearman rank-order correlations among the main variables are presented in Table 3.

Parental anxiety is positively associated with both parents’ and children’s physiological arousal levels. Specifically, higher parental anxiety was related to a higher mean HR (ρ = 0.419, *p* < 0.05), suggesting increased physiological activation in more anxious parents. In addition, parental anxiety was significantly associated with a higher mean heart rate (ρ = 0.512, *p* < 0.01) and a higher child HR/RMSSD ratio (ρ = 0.428, *p* < 0.05). This pattern indicates that parental anxiety may extend beyond the individual and may be linked to elevated arousal and reduced autonomic regulatory balance in children.

However, parental anxiety was not significantly associated with HRV indices (SDNN and RMSSD) in either parents or children, suggesting that anxiety primarily influences general arousal rather than the parasympathetic regulation.

A coherent autonomic pattern emerged within the children’s physiological measures. A higher mean HR was strongly associated with a lower SDNN (ρ = −0.747, *p* < 0.001) and RMSSD (ρ = −0.826, *p* < 0.001), indicating that increased arousal is coupled with reduced HRV and diminished parasympathetic regulation. Furthermore, the HR/RMSSD ratio was strongly positively associated with the mean heart rate (ρ = 0.958, *p* < 0.001) and strongly negatively associated with both SDNN (ρ = −0.838, *p* < 0.001) and RMSSD (ρ = −0.934, *p* < 0.001). This supports the interpretation of the HR/RMSSD ratio as an index of heightened arousal and reduced regulatory capacity.

Overall, these findings suggest that children’s physiological responses reflect a tightly coupled arousal–regulation system and that parental anxiety may shape children’s physiological arousal within dyadic interactions.

**Figure 1:**
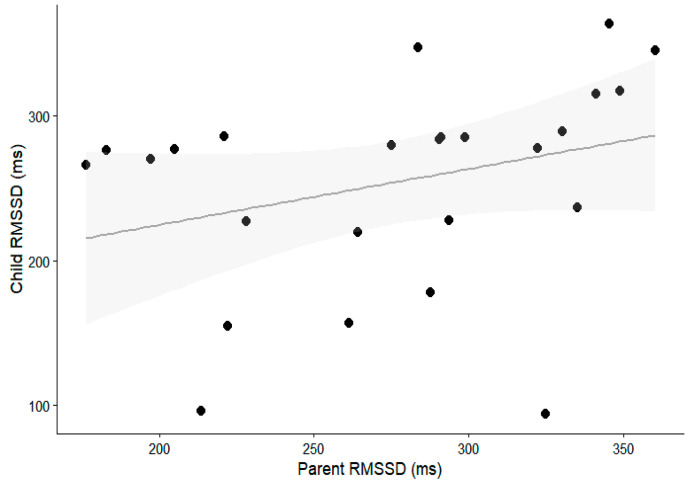
Association between parent and child RMSSD during free-play interaction.

To further illustrate the association between the parent and child RMSSD during free-play interactions, a scatterplot is presented in Figure 1. Parent RMSSD was positively associated with child RMSSD, indicating that higher parasympathetic activity in parents was associated with higher parasympathetic activity in their children. The linear model accounted for a small proportion of the variance (R^2^ = 0.093), suggesting a modest association with considerable variability across dyads.

### 3.3. Group Differences by Attachment Security

Group differences in physiological indices according to attachment security are presented in Table 4. Independent samples *t*-tests (with Welch’s correction where appropriate) were conducted to compare children with secure and resistant attachment.

Significant differences were observed in the mean heart rates of the children. Children with resistant attachment exhibited a significantly higher mean HR (M = 110.48, SD = 11.14) than those with stable attachment (M = 93.93, SD = 14.63), t(23) = 2.69, *p* = 0.013, with a large effect size (Hedges’ g = 1.16). This suggests that children with resistant attachments exhibit higher physiological arousal.

For HRV, children with resistant attachment showed a lower RMSSD (M = 203.85, SD = 87.33) than those with stable attachment (M = 273.51, SD = 55.47), indicating reduced parasympathetic regulation. However, this difference was not statistically significant (t(7.96) = −1.96, *p* = 0.086), despite a large effect size (Hedges’ g = −1.03).

Similarly, the HR/RMSSD ratio was higher in the resistant-attachment group (M = 0.69, SD = 0.41) than in the stable-attachment group (M = 0.37, SD = 0.14), suggesting greater physiological arousal relative to regulatory capacity. However, this difference was not statistically significant, t(6.53) = 1.99, *p* = 0.089, although the effect size was large (Hedges’ g = 1.27).

Overall, these findings indicate that children with resistant attachment tended to exhibit higher physiological arousal and lower parasympathetic regulation than those with stable attachment. However, except for the mean HR, these differences did not reach statistical significance, likely due to the small sample size despite the large effect sizes.

## 4. Discussion

The present study examined the association between parent and child physiological synchrony and children’s attachment-related physiological responses during parent–child interactions. A key finding of this study was the positive association between parent and child RMSSD during free-play interactions, suggesting coordinated parasympathetic activity within parent–child dyads. This pattern is consistent with the notion of physiological synchrony as a normative dyadic process that emerges during everyday interactions, independent of attachment classifications. Overall, the findings support the view that attachment is not merely a behavioral classification but a developmental system encompassing emotional and autonomic nervous system functioning under stress. Early attachment has a small but reliable influence on later socioemotional development, serving as a foundational factor that accumulates across diverse developmental pathways. Secure attachment is associated with more positive social relationships, better emotion regulation, and adaptive behavior, whereas insecure attachment is more closely linked to problem behaviors, emotional difficulties, and relationship problems (Groh et al., 2017). In addition, attachment is related to children’s behavioral and emotional responses in stress-related contexts, indicating a link between attachment and patterns of stress-related regulation (Ainsworth et al., 1978; Bowlby, 1969/1982).

First, regarding the associations between variables, parental anxiety was positively associated with the mean HR of the parents, indicating that higher anxiety levels were linked to increased physiological arousal in parents. Given that HR is a well-established index of sympathetic activation, this finding suggests that parental anxiety is reflected in heightened physiological activation. Importantly, parental anxiety was significantly associated with children’s physiological indices, including a higher mean HR and HR/RMSSD ratio. This pattern indicates that parental anxiety may extend beyond the individual and may be linked to increased physiological arousal and reduced autonomic balance in children. These findings suggest that parental anxiety may be associated with children’s physiological arousal within dyadic interaction contexts, supporting theoretical perspectives that emphasize emotional transmission and shared physiological processes (Perlman et al., 2022; C. G. Smith et al., 2022).

Simultaneously, the absence of significant associations between the HRV indices (SDNN and RMSSD) suggests that parental anxiety may be more closely related to overall arousal than to parasympathetic functioning. In addition, a significant positive association was observed between the RMSSD of parents and children, indicating a link between the parasympathetic activities of parents and children during free-play interactions.

RMSSD is widely used as an index of vagally mediated parasympathetic activity (Shaffer & Ginsberg, 2017), and is included among standard HRV metrics defined by established guidelines (Task Force, 1996). Thus, the present findings indicate that parent and child RMSSD values are positively associated, suggesting that parasympathetic activity tends to co-vary across different interactions. Notably, this association emerged for RMSSD but not for mean HR, suggesting that such covariation may be more evident in parasympathetic processes than in overall arousal levels. This pattern is consistent with theoretical perspectives that conceptualize the parent–child relationship as involving physiological synchrony (Feldman, 2007, 2012), as well as with empirical studies demonstrating that synchrony can be observed across diverse interaction contexts and measured using a range of methodological approaches (Atzil et al., 2014; Davis et al., 2018; Lunkenheimer et al., 2015). In particular, previous research has shown that physiological synchrony can be observed in both naturalistic and structured parent–child interactions (Miller et al., 2019; Wass et al., 2019; Lunkenheimer et al., 2015), supporting the interpretation that such coordination represents a common, though context-dependent, feature of early social interaction. The present findings extend this literature by showing that such coordinated patterns can also be detected using summary indices of parasympathetic activity (e.g., mean RMSSD) computed over the interaction periods. Moreover, evidence from adult interaction contexts indicates that physiological synchrony can emerge even between unfamiliar individuals (Zeevi et al., 2022), suggesting that synchrony may reflect a broader mechanism underlying social connection rather than a process specific to attachment relationships. However, these findings should be interpreted with caution. Synchrony is inherently a temporal and dynamic process and, therefore, cannot be fully captured by correlational analyses of aggregated indices. Accordingly, the present results are best understood as reflecting patterns consistent with physiological synchrony rather than as direct evidence of synchrony itself.

Second, regarding group differences, children with resistant attachment exhibited a significantly higher mean HR than those with secure attachment, indicating heightened physiological arousal. Although children with resistant attachment also showed lower RMSSD and higher HR/RMSSD ratios than children with secure attachment, these differences were not statistically significant, despite relatively large effect sizes. This pattern suggests a tendency toward reduced parasympathetic functioning and greater autonomic imbalance in resistant attachment; however, the evidence remains preliminary. The lack of statistical significance may be attributable to the limited statistical power, given the small sample size. These findings are broadly consistent with attachment theory (Ainsworth et al., 1978; Bowlby, 1969/1982).

Simultaneously, the present results suggest that such differences may be more robustly reflected in general physiological arousal (HR) than in parasympathetic indices. These findings are partially consistent with those of previous psychophysiological studies (e.g., Hill-Soderlund et al., 2008; J. D. Smith et al., 2016). Importantly, this study extends previous research by demonstrating these patterns in a context that integrates naturalistic interactions with stress-related episodes.

Taken together, the findings of this study suggest that parent–child physiological responses exhibit coordinated patterns during interactions, which is consistent with the concept of physiological synchrony. These patterns appear to emerge in everyday interaction contexts, regardless of attachment classification, whereas attachment-related differences are more clearly reflected in physiological arousal under stress conditions. These findings suggest that physiological synchrony may reflect a fundamental dyadic regulatory mechanism that extends beyond typical interaction contexts.

The clinical significance of these dyadic regulatory processes is further underscored by research in high-risk populations, such as preterm newborns. For instance, La Rosa et al. (2024) highlight how affective touch—mediated by C-tactile fibers—serves as a primary neurobiological mechanism for establishing early co-regulation and attachment. Just as our findings show that RMSSD synchrony supports regulation in toddlers, early tactile interventions like Skin-to-Skin Contact (SSC) or Kangaroo Care may provide the foundational physiological stability necessary to mitigate the developmental risks associated with preterm birth and NICU-related sensory deprivation.

### 4.1. Theoretical Contribution

This study contributes to the literature by demonstrating that physiological synchrony, as reflected in RMSSD covariation, may function as a shared dyadic process that operates regardless of attachment classification. Importantly, this finding extends prior attachment research by emphasizing normative interactional processes that co-occur across parent–child dyads rather than focusing solely on individual differences. Thus, the present study provides an integrative perspective from which attachment emerges from both shared physiological processes and individual variability.

### 4.2. Limitations

Despite its contributions, this study has several limitations. First, the relatively small sample size may have limited statistical power, particularly in detecting group differences in parasympathetic indices. Second, synchrony was inferred from correlational analyses of aggregated indices that did not capture their temporal and dynamic nature. Third, this study was conducted in a specific context that may have limited the generalizability of the findings.

### 4.3. Future Research Directions

Future research should incorporate time-series and dynamic analytical approaches to examine physiological synchrony more directly. Longitudinal designs are also valuable for understanding how synchrony- and attachment-related physiological patterns codevelop over time. In addition, expanding research to diverse populations and contexts may help clarify the generalizability of these findings.

### 4.4. Implications

These findings may have implications for the early identification and intervention in populations at risk of disrupted parent–child interactions, such as preterm infants. Interventions that support parent–child interactions may facilitate adaptive physiological functioning, although these implications should be interpreted with caution, given the correlational nature of the present data.

## 5. Conclusions

This study examined the relationships among parental anxiety, parent–child physiological synchrony, and children’s attachment-related physiological responses within a parent–child interaction context. The results showed that parental anxiety was associated with increased physiological arousal. In addition, parental anxiety was significantly related to children’s physiological indices, including a higher mean HR and HR/RMSSD ratio, suggesting that parental emotional states may be reflected in physiological arousal and autonomic balance. These findings indicate that the influence of the parental emotional state on children’s physiological responses may be directly observed in their concurrent physiological responses. At the dyadic level, a significant positive association was observed between parents and children in RMSSD, suggesting covariation in parasympathetic activity within parent–child dyads, consistent with physiological synchrony during interaction.

In addition, significant group differences were observed only in the mean HR. Children with resistant attachment exhibited a higher mean HR than those with secure attachment, reflecting heightened physiological arousal. Although children with resistant attachment also showed lower RMSSD and higher HR/RMSSD ratios, these differences were not statistically significant, suggesting that evidence for differences in parasympathetic functioning is limited. These findings suggest that attachment security is associated with differences in children’s physiological arousal under stress-related conditions, particularly in terms of overall arousal.

Overall, the present findings indicate that children’s physiological responses are associated with emotional and physiological processes in parent–child interactions. In particular, the significant associations between parental anxiety and children’s physiological arousal, as well as the observed parasympathetic covariation between parents and children, highlight the role of parental emotional functioning and dyadic interactions in shaping children’s physiological responses. Importantly, these findings suggest a shared dyadic regulatory process that may operate regardless of the attachment classification. Repeated interactive experiences may be related to the development of attachment-related patterns over time. These findings highlight the importance of understanding attachment as a process that gradually develops within parent–child relationships through ongoing interactions involving both shared physiological processes and individual differences.

## Figures and Tables

**Table 1:** Demographic Characteristics of Participants (N = 25).

Variable	n (%) or M (SD)
Child age (months)	36.48 (5.21)
Parent age (years)	37.30 (4.14)
Child sex	
Male	9 (36.0%)
Female	16 (64.0%)
Parent sex	
Father	3 (12.0%)
Mother	22 (88.0%)
Parent education	
High school graduate	2 (8.0%)
College graduate	23 (92.0%)
Household economic status	
Upper-middle	8 (32.0%)
Middle-middle	13 (52.0%)
Lower-middle	4 (16.0%)

**Table 2:** Descriptive statistics of physiological and psychological variables (N = 25).

Variable	M	SD	Min	Max
Parent mean HR (bpm)	73.75	9.79	58.95	95.32
Child mean HR (bpm)	98.57	15.50	55.22	121.28
Parent SDNN (ms)	296.61	75.26	153.36	416.18
Child SDNN (ms)	341.47	103.90	116.84	501.50
Parent RMSSD (ms)	276.03	56.33	176.27	360.32
Child RMSSD (ms)	254.01	71.45	94.16	363.08
Child HR/RMSSD ratio	0.46	0.28	0.15	1.29
Parent anxiety	3.16	2.25	0.00	7.00

**Table 3:** Spearman correlations among main variables.

Variable	1	2	3	4	5	6	7
1 Parent mean HR							
2 Child mean HR	0.359 †						
3 Parent SDNN	−0.272	−0.292					
4 Child SDNN	−0.333	−0.747 ***	0.275				
5 Parent RMSSD	−0.355 †	−0.212	0.948 ***	0.263			
6 Child RMSSD	−0.282	−0.826 ***	0.505 **	0.828 ***	0.477 *		
7 Child HR/RMSSD ratio	0.356 †	0.958 ***	−0.365 †	−0.838 ***	−0.320	−0.934 ***	
8 Parent anxiety	0.419 *	0.512 **	0.100	−0.344 †	0.110	−0.304	0.428 *

*** *p* < 0.001, ** *p* < 0.01, * *p* < 0.05, † *p* < 0.1.

**Table 4:** Group differences in physiological variables by attachment security.

Variable	Stable Attachment M (SD)	Resistant Attachment M (SD)	t (df)	*p*	Hedges’ g
Child mean HR (bpm)	93.93 (14.63)	110.48 (11.14)	2.69 (23)	0.013	1.16
Child RMSSD (ms)	273.51 (55.47)	203.85 (87.33)	−1.96 (7.96)	0.086	−1.03
Child HR/RMSSD	0.37 (0.14)	0.69 (0.41)	1.99 (6.53)	0.089	1.27

## Data Availability

The data are not publicly available due to privacy and ethical restrictions but are available from the corresponding author upon reasonable request.

## Abbreviations

The following abbreviations are used in this manuscript:

HR	Heart Rate
HRV	Heart Rate Variability
RMSSD	Root Mean Square of Successive Differences
SDNN	Standard Deviation of Normal-to-Normal Intervals
